# Comparison of immediate versus delayed streak plate inoculation on urine bacterial culture and susceptibility testing in dogs and cats

**DOI:** 10.1111/jvim.15719

**Published:** 2020-01-31

**Authors:** Emily L. Coffey, Kim Little, Davis M. Seelig, Aaron K. Rendahl, Jennifer L. Granick

**Affiliations:** ^1^ Department of Veterinary Clinical Sciences, College of Veterinary Medicine University of Minnesota Saint Paul Minnesota

**Keywords:** delayed culture, quantitative bacterial culture, urinary tract infection, urine culture

## Abstract

**Background:**

Quantitative bacterial culture and susceptibility testing is the gold standard diagnostic for determining bacterial urinary tract infection. Transport of samples to external reference laboratories is common practice in veterinary medicine.

**Objective:**

To compare bacterial culture and susceptibility results from clinical urine samples when streak plate inoculation is performed immediately after sample collection versus after transport to a reference laboratory. To determine the clinical implications of discrepant culture results.

**Animals:**

One hundred and ninety‐four canine and 45 feline urine samples that were submitted for urinalysis and urine culture and susceptibility testing.

**Methods:**

This was a prospective, cross‐sectional study. Streak plate inoculations were performed on urine samples immediately after collection and also after transport to a reference laboratory. Samples were stored in plain sterile tubes and refrigerated up to 24 hours before transport. Culture results were compared, and discordant results were evaluated for clinical relevance. Signalment, comorbidities, lower urinary tract signs, and antimicrobial history were recorded.

**Results:**

Kappa coefficient for agreement between plating methods was 0.884. Twenty‐two (71%) of 31 discrepant results were determined to have no clinical impact. Though 35% of clean midstream samples had discrepant culture results, only 8% of these had clinical impact. Conversely, 8.6% from cystocentesis were discrepant, but 41% of these had clinical impact.

**Conclusions and Clinical Importance:**

Provided urine samples are stored and transported appropriately, the immediate preplating of urine for culture and susceptibility testing is unnecessary in the majority of cases. Despite more discrepancies in plating methods for midstream samples, the minority were of clinical importance.

**Abbreviations**
CFU: colony‐forming units
CI: confidence interval
HPF: high‐powered field
ISCAID: International Society of Companion Animal Infectious Diseases
LUT: lower urinary tract
MIC: minimum inhibitory concentration
QBC: quantitative bacterial culture
UTI: urinary tract infection
WBC: white blood cell

## 1 INTRODUCTION

The emergence of antimicrobial resistance has prompted considerable discussion in the veterinary community regarding responsible antimicrobial usage and culture‐guided treatment. Genitourinary tract infections are a common indication for antimicrobial treatment in small animals, representing approximately 12% of all PO antibiotic prescriptions in dogs.^1^, ^2^ Unfortunately, antimicrobials are commonly prescribed despite lack of confirmed diagnosis.^3^ Quantitative bacterial culture (QBC) and susceptibility testing with concurrent urinalysis are the gold standard for diagnosing infection and selecting treatment. Cost is the most common barrier cited by veterinarians to performing QBC.^4^ When a urine QBC is recommended, veterinarians and owners should have assurance of the reliability and diagnostic utility of the test. Studies evaluating the impact of a delay in urine culture processing have been primarily performed in institutions with on‐site bacteriology laboratories. However, transportation of urine samples to outside reference laboratories is the norm for most primary care settings, and the effects of both a delay in culture inoculation and transportation to reference laboratories on QBC and susceptibility results have not been evaluated.

Immediate inoculation of culture media with urine is recommended for optimal diagnostic accuracy of QBC.^5^ However, transport to external laboratories can delay media inoculation by 24 hours or more.^5^, ^6^, ^7^ Studies evaluating the impact of storage on canine urine samples have yielded variable results. Though storage at 4°C is recommended if immediate processing is unavailable, some studies have shown a decreased sensitivity of bacterial recovery and an increased (4%) false‐negative rate with delayed inoculation despite refrigeration.^5^, ^6^, ^7^

Currently, veterinary studies evaluating the impact of delay of urine culture processing are restricted to experimentally inoculated samples or to clinical samples collected via cystocentesis. Guidelines created by the International Society of Companion Animal Infectious Diseases (ISCAID) recommend cultures are preferentially performed on cystocentesis samples, though other studies have demonstrated that voided samples can yield accurate results.^8^, ^9^, ^10^ The guidelines recommend urine samples with delayed processing are refrigerated and plated for culture within 24 hours of collection.^9^, ^10^ However, there are no studies to confirm that such recommendations, when applied to clinical samples of various collection techniques, preserve the accuracy of QBC testing following transport to an external laboratory. There is also no investigation into how altered results secondary to delayed processing could impact clinical decision‐making.

The primary objective of this study was to compare QBC and susceptibility results from clinical canine and feline urine samples obtained via variable collection techniques when a streak plate inoculation was performed immediately after sample collection or performed following transport to a reference laboratory. We hypothesized that QBC results differ between samples with delayed versus immediate inoculation, both in bacterial species and concentration in colony‐forming units (CFU)/mL. A secondary objective was to determine whether such differences would impact decisions regarding antimicrobial treatment.

## 2 MATERIALS AND METHODS

This was a prospective, cross‐sectional study. Urine samples from dogs and cats submitted for routine urinalysis and aerobic QBC and susceptibility to the clinical pathology laboratory at the University of Minnesota Veterinary Medical Center were included. Two‐hundred samples were collected from October 2016 to March 2017. However, initial data analysis revealed that 5 additional samples collected via midstream catch and 32 collected via cystocentesis were needed to determine differences between these 2 collection methods. For this reason, urine sample collection was resumed in October 2017. To be included, a minimum of 10 μL of urine must have been available after diagnostics requested by the attending clinician were completed. A urinalysis was required at the time of culture to be included, and urine from any collection method could be included. All samples, including clean midstream samples, were collected in hospital. According to the University of Minnesota's Institutional Animal Care and Use Committee, this study was exempt from formal ethical approval. Written owner consent was not needed, as all samples were acquired during routine diagnostic investigation, and no additional samples were obtained or interventions performed for the purpose of the study.

Samples were submitted in sterile, preservative‐free, plastic tubes (Greiner Bio‐One, Vacuette Tube, Kremsmünster, Austria). Aliquots of urine samples were transferred to Blood Agar Plates followed by transfer to MacConkey (Remel, ThermoFisher Scientific, Waltham, Massachusetts) by using a conventional streaking technique for quantitative colony isolation. Briefly, the plates were labeled with the dog or cat's information, date, and loop size. The loop was dipped into thoroughly mixed urine, and a primary streak was made down the center of the blood agar. With the side of the loop, the plate was streaked by means of a back‐and‐forth motion.^11^ Ten microliter aliquots were inoculated for samples collected via cystocentesis, feline clean midstream catch, or feline catheterization. One microliter aliquots of urine were inoculated for canine clean midstream catch or canine catheterization specimens in accordance with laboratory protocol.^11^, ^12^ Plates were incubated aerobically at 35°C before transport to an outside reference laboratory (Marshfield Labs, Marshfield, Wisconsin). The remainder of the sample was refrigerated at 2°C‐8°C while awaiting transport to the same reference laboratory. Culture plates were transported at room temperature, and urine specimens awaiting plate inoculation were transported in a refrigerated pack within 24 hours of sample collection. Approximate travel time to the reference laboratory was 5‐6 hours after retrieval from the primary institution. Specimens were plated upon arrival to the reference laboratory by using the same protocol. Culture media was evaluated for growth at 24 hours for all samples, and again at 48 and 72 hours for samples collected via cystocentesis. Minimum inhibitory concentrations (MICs) of isolates were determined by broth dilutions (Sensititre, ThermoFisher Scientific, Waltham, Massachusetts). Susceptibility breakpoint interpretations were based on Clinical Laboratory Standards Institute guidelines.^13^, ^14^

The corresponding medical records were reviewed. The dog or cat's sex, species, and age at the time of sample collection were recorded. The presence or absence of clinical signs localizing to the lower urinary tract (LUT) was recorded. Clinical signs were defined as stranguria, hematuria, dysuria, malodorous urine, pollakuria, periuria, and urinary incontinence. The administration of any antibiotic within 3 days of sample collection was noted, but these samples were not excluded in the event that antibiotic administration impacted the growth on 1 plating method differently than the other. The presence of endogenous or exogenous immunosuppression was recorded and categorized as diabetes mellitus, hyperadrenocorticism, or iatrogenic from immunosuppressive medications. Immunosuppressive medications included chemotherapeutic agents, corticosteroids, cyclosporine, azathioprine, mycophenolate mofetil, or leflunomide. For corticosteroids to be considered immunosuppressive, the dose must have been ≥1 mg/kg/day. Urinalyses were performed at the same time as urine culture collection. White blood cells (WBCs) in urine sediment were recorded as follows: No WBC/high‐powered field (HPF) (score 0); occasional WBC/HPF (score 1); 0‐5 WBC/HPF (score 2); 5‐20 WBC/HPF (score 3); 20‐50 WBC/HPF (score 4); >50 WBC/HPF (score 5). These scoring categories were in accordance with institutional laboratory protocol for WBC/HPF reporting. Bacteriuria was recorded as none, few, moderate, or many bacteria per HPF, according to standardized laboratory protocol.

The QBC results were categorized as negative, minimal growth, or marked growth according to previously recommended definitions.^9^, ^15^ If obtained via cystocentesis, bacterial growth ≥10^3^ CFU/mL was considered to be marked growth. For samples collected via catheter, bacterial counts ≥10^4^ CFU/mL in males and ≥10^5^ in females were considered to be marked growth. For samples collected via clean midstream catch, ≥10^5^ CFU/mL in dogs and ≥10^4^ CFU/mL in cats were considered to be marked growth. Any sample with positive bacterial growth but with a bacterial concentration below the criteria for infection, as determined by these definitions, was labeled as minimal growth.

Discrepancies in QBC and susceptibility results were defined as any difference in organism(s) cultured, the bacterial concentration, or the results of the susceptibility panel. A discrepancy was determined to have a clinical impact if differences were present in antibiotic susceptibility profile or bacterial species cultured, as well as if bacterial growth was considered negative or minimal growth with 1 plating method but marked growth with the other. However, if differences in susceptibility profile were within a single MIC dilution, this was not considered to have a clinical impact.

### 2.1 Statistical analysis

Percent disagreement and percent clinical impact (for those that disagreed) were compared by collection method. Weighted and unweighted kappa agreement was calculated between QBC growth categories (negative, minimal growth, marked growth) of immediate and delayed culture methods. Weighted kappa agreement treated negative‐versus‐marked growth discrepancies as a stronger disagreement than negative‐versus‐minimal growth or minimal‐versus‐marked growth discrepancies. Percent infection by WBC category (<3 and ≥3) as well as percent of LUT signs by WBC category (<3 and ≥3), both including and excluding those with WBC score of 0, were compared. Percent agreement by whether antibiotics were used or if the dog or cat was immunocompromised was evaluated, and the percent of immediate growth by immunocompromised status, LUT signs, and antibiotic usage were compared. Percentages are reported with 95% confidence intervals (CIs), by using the Agresti‐Coull method,^16^ and differences are tested for by using the chi‐squared test with the N‐1 correction.^17^ All analyses performed in R version 3.5.1 (July 2, 2018; R Core Team 2018).^18^

## 3 RESULTS

### 3.1 Study population

Aerobic bacterial cultures were performed on a total of 239 urine samples that fit the inclusion criteria, 45 (45/239, 18.8%) from cats and 194 (194/239, 81.2%) from dogs. The samples were obtained by cystocentesis (197/239, 82.4%), clean midstream catch (37/239, 15.5%), and urethral catheterization (5/239, 2.1%). Population data are presented in Table 1.

**Table 1:** Population characteristics of dogs and cats meeting inclusion criteria

	Dog (n = 194)	Cat (n = 45)
Age (years)	7.8 (0.25‐15)	9.6 (0.67‐21)
Male (N/I)	56 (47/9)	22 (22/0)
Female (S/I)	138 (121/17)	23 (23/0)
Cystocentesis	153	44
Catheterized	4	1
Free catch	37	0

*Note*: Age is reported as median (range).

Abbreviations: I, intact; N, neutered; S, spayed.

### 3.2 Bacterial culture results

Of all urine samples, 28% (67/239 for immediate plating, 68/239 for delayed plating) were positive for bacterial growth. The majority of positive cultures on both immediate (59/67, 88%) and delayed (55/68, 81%) plating methods grew a single organism. The most common organisms cultured were *Escherichia coli* (36/76, 47%), followed by *Proteus mirabilis* (11/76, 15%), *Staphylococcus* spp. (6/76, 8%), *Klebsiella pneumonia* (5/76, 7%), and *Enterococcus* spp. (5/76, 7%) (Table 2). Clinical signs consistent with LUT disease were present in 103 dogs and cats. Twenty‐nine percent (30/103, 95% CI 21.2%, 38.6%) of dogs and cats with LUT signs had marked growth on culture via immediate plating versus 13.2% (17/129, 95% CI 8.3%, 20.2%) that lacked LUT signs (Table 3). There was a significant correlation between the presence of LUT signs and the presence of marked bacterial growth (*P* < .01). Of animals with LUT signs but without marked bacterial growth, there was no difference between dogs and cats. Twenty‐four of the 129 dogs and cats without LUT signs had positive QBC, 17 of which had marked bacterial growth. Antibiotic administration within 3 days of urine collection was identified in 39 dogs and cats (39/239, 16.3%) and immunosuppression was identified in 67 (67/239, 28.0%). There was no association between immunosuppressive status and any level of bacterial growth (*P* = .58) or between antibiotic administration and any level of bacterial growth (*P* = .23).

**Table 2:** Bacterial isolates identified in canine and feline urinary tract infections classified by immediate or delayed plating method, as well as by minimal or marked growth classification

Organism	Immediate		Delayed	
	Minimal	Marked	Minimal	Marked
*Escherichia coli*	7	29	4	35
*Proteus mirabilis*	3	8	1	10
*Staphylococcus* spp.	2	4	3	6
*Enterococcus* spp.	1	4	0	5
*Klebsiella pneumonia*	1	4	1	4
*Streptococcus* spp.	2	2	1	1
*Enterobacter cloacae*	0	3	0	2
Mixed LUT flora	3	0	1	0
*Arcanobacterium* spp	1	0	0	1
*Coryneform bacilli*	1	0	1	0
Gram‐negative bacilli	1	0	4	1
Gram‐positive cocci	0	0	1	0

*Note*: Organisms are listed in descending order of positive immediate culture results.

Abbreviation: LUT, lower urinary tract.

**Table 3:** Pyuria, bacteriuria, and presence of clinical signs of lower urinary tract disease in relation to bacterial culture results

	Negative (n = 172)	Minimal (n = 18)	Marked (n = 49)
WBC score			
0‐2	164 (95.3%)	10 (55.6%)	15 (30.1%)
3‐5	8 (4.7%)	8 (44.4%)	34 (69.4%)
Cytologic bacteriuria (per HPF)			
None	167 (97.1%)	12 (66.7%)	3 (6.1%)
Few	4 (2.3%)	3 (16.7%)	4 (8.2%)
Moderate to many	1 (0.6%)	3 (16.7%)	42 (85.7%)
LUT signs			
Yes	62 (36.0%)	11 (61.1%)	30 (61.2%)
No	105 (61.0%)	7 (38.9%)	17 (34.7%)
Unknown	5 (2.9%)	0 (0%)	2 (4.1%)

*Note*: WBC score 0‐2 is equivalent to 0‐5 WBC/HPF; WBC score 3‐5 is equivalent to 5 to >50 WBC/HPF. Culture results are based on immediate plating method.

Abbreviations: HPF, high powered field; LUT, lower urinary tract; WBC, white blood cells.

### 3.3 Immediate versus delayed plating

Identical QBC and susceptibility results were noted in 87.0% (208/239) of samples. The unweighted kappa agreement was 0.884, and the weighted kappa agreement was 0.921. Among the 31 (31/239, 12.9%) discrepancies, differences in type and number of organisms cultured, quantification of bacteria (CFU/mL), and results of the antibiotic susceptibility profile were noted (Table 4). Quantitative bacterial counts or the number of bacterial species isolated were higher in the immediate cultures in 8/29 cases (28%; 95% CI 14.5%, 45.9%) but higher in delayed cultures in the remaining 21 samples (*P* = .03). *Proteus mirabilis* was specifically evaluated because of its tendency to swarm across agar plates. Only 20% (1/5) of *P. mirabilis* discrepancies were attributable to higher growth with immediate plating, versus 25% (7/28) of non‐*P. mirabilis* species. Similarly, we found no pattern between discrepant growth and any particular bacterial species. The remaining 2 samples did not have differences in the amount or type of organism cultured, but differences in the antibiotic susceptibility profiles. Any changes in how the susceptibility profiles were reported was considered a discrepancy, regardless of whether they were considered to have a clinical impact. In 1 sample, *E. coli* was reported to have an intermediate interpretation to amikacin on the immediately plated aliquot (MIC ≤4 μg/mL), but a susceptible interpretation to amikacin on delayed plating (MIC 8 μg/mL). In another sample, *E. coli* cultured from an immediately plated aliquot was interpreted as resistant to orbifloxacin, whereas the delayed‐plating aliquot was given an intermediate interpretation (MIC 8 and 4 μg/mL, respectively). However, given that the difference in MIC was only 1 dilution apart, this change was not deemed clinically relevant. Neither antibiotic usage nor immunosuppression significantly impacted agreement between plating methods (*P* = .94 and *P* = .35 respectively).

**Table 4:** Discrepancies between plating methods

	Higher growth in immediate (n = 8)	Higher growth in delayed (n = 21)
Discordant (CFU/mL)	1	8
Discordant bacterial species and/or strains	7	13
Discordant susceptibility profile	2	

*Note*: Higher growth indicates larger quantitative bacterial counts (CFU/mL) or more bacterial species isolated.

Twenty‐two (71%) of the 31 discrepancies were deemed to have no clinical impact, but 9 (29%) were deemed to have clinical relevance (Table 5). Cases that were collected via clean midstream catch were more likely to have discrepancies between plating methods than those collected via cystocentesis (*P* < .001). Of the 17 cystocentesis samples that had disagreement between plating methods, 7 had a clinical impact (41%; 95% CI 21.6%, 64.0%). However, of the 13 clean midstream catch samples that disagreed, only 1 of these disagreements would have had a clinical impact (8%; 95% CI 0%, 35.4%). Thus, although cases that were collected via clean midstream catch were more likely to have disagreement in culture results, there was higher likelihood of a clinical impact of these discrepancies in the cystocentesis samples (*P* = .04). The 1 discrepant catheterized sample was deemed to have clinical impact.

**Table 5:** Agreement of bacterial culture and susceptibility results between immediate and delayed plating methods and clinical impact of discrepant results

Collection method	Total urine samples (n = 239)	# Discordant samples (n = 31)	% Discordant samples	% Discordant samples within collection method	% Discordant samples with clinical impact (n = 9)
Cystocentesis	197	17	55% (17/31)	8.6% (17/197)	41% (7/17)
Free catch	37	13	42% (13/31)	35% (13/37)	8% (1/13)
Catheter	5	1	3% (1/31)	20% (1/5)	100% (1/1)

### 3.4 Urinalysis results

There were significantly lower WBC scores (score of 0‐2) in negative culture samples, and significantly higher scores (scores 3‐5) in samples with marked bacterial growth (*P* < .001) (Table 3). Eliminating samples with no detectable WBCs, samples with WBC scores of 1‐2 were still significantly less likely to have marked bacterial growth (*P* < .001). In urine samples in which WBC scores >3, dogs and cats were twice as likely to exhibit LUT signs than in those with lower WBC scores (35/48, 73% with WBC score of 3‐5 versus 68/184, 36.9% with WBC score of 0‐2). Of dogs and cats lacking LUT signs but with QBC results that demonstrated marked bacterial growth, 9 of 17 had WBC scores of 3 or greater; 1 additional dog had a WBC score of 2 and was the only 1 of these 17 that lacked cytologic bacteriuria. Of the 17 dogs and cats with positive QBC lacking LUT signs, 2 were diabetic and 2 were receiving immunosuppressive medications; 3 of these 4 had WBC scores of 4 or greater.

Cytologic bacteriuria was present in 23.8% of all cases (57/239). Most urine samples (167/182, 91.8%) without bacteriuria had negative growth via immediate culture, whereas only 3 had marked bacteriuria (Table 3). Similarly, most (46/57, 81%) samples in which bacteria were identified in urine sediment had marked bacterial growth. Of the 5 cases with negative cultures and positive cytologic bacteriuria, 4 had “few” bacteria reported per HPF in urine sediment. The remaining sample had “moderate” rods and “few” coccoid bacteria on cystocentesis urinalysis with a negative culture. This dog had no LUT signs, a WBC score of 1, and no history of immunosuppression or antibiotic use within 3 days of sample collection.

## 4 DISCUSSION

Urinary tract disease is a common indication for antimicrobial prescribing in small animal veterinary practices. However, given the cost of QBC and financial limitations of owners, urinary tract infection (UTI) diagnoses are inconsistently confirmed.^4^ Routine use of QBC and susceptibility testing in dogs and cats with suspected UTIs supports antimicrobial stewardship by guiding effective treatment and allowing veterinarians to monitor for antimicrobial resistance. This study sought to verify that common methods of sample handling and transport do not alter results or clinical utility of urine QBCs. The majority of private veterinary practices depend upon transportation of urine cultures to outside reference laboratories. However, few studies have evaluated whether the delay and transport impacts culture results of clinical samples, if discrepancies would impact clinical decision‐making, or if the type of sample collection affects agreement between plating methods. This study found that there was a strong agreement between QBC results from immediate and delayed plating.

Among samples with discrepancies between plating methods, there was commonly more bacterial growth among delayed‐plating aliquots, which could be due to overgrowth of the primary pathogen, contaminant during storage and transport, or both. Of the discrepant results, 71% (22/31) of those differences were not considered to have affected decisions regarding antimicrobial prescribing. Changes that defined clinical impact included those that would impact whether treatment was indicated (bacterial concentration) or alter antimicrobial selection (type of organism cultured or susceptibility profile results). Based on these criteria, less than 4% (9/239) of all samples had discrepant culture results that would have affected clinical decision‐making.

In the current study, clean, midstream catch samples were significantly more likely to have disparate culture results compared to samples collected via cystocentesis, though these discrepancies had minimal clinical impact. Current guidelines recommend avoiding urine culture and susceptibility testing on voided samples.^10^ However, when using a cutoff value of >100 000 CFU/mL to define marked bacteriuria, voided urine samples can yield accurate QBC results.^8^ In this study, only 15.5% (37/239) of total samples were collected via clean midstream catch, yet these samples represented 42% (13/31) of all discordant results. However, only 8% (1/13) of these disparate results were deemed to impact clinical decision‐making. Samples collected via cystocentesis represented 82.4% (197/239) of total urine samples, and 55% (17/31) of the discrepant cultures, half of which were determined to have clinical impact. We found no pattern in overgrowth of contaminants or loss of viable organisms among collection methods. Voided urine samples had minimal clinically relevant discrepancies between culture methods, suggesting that if using the recommended cutoff of 100 000 CFU/mL, clean midstream catch samples can be an acceptable form of sample collection.

This study was consistent with previous findings regarding the association of pyuria with bacteriuria. Degree of pyuria was significantly, positively associated with marked bacterial growth in this study. Only 7.9% (15/189) of urine samples with pyuria scores of 0‐2 had marked growth on QBC compared to 68% (34/50) of those with pyuria scores greater than 2. These findings support past studies that pyuria is common in dogs with suspected UTIs.^19^ Veterinarians often treat empirically with antimicrobials in dogs and cats with pyuria and LUT signs. However, LUT signs were common among those in this study population with pyuria, regardless of whether or not bacteriuria was present. There was also a significant correlation between the presence of LUT signs and marked bacteriuria, though LUT signs alone could not accurately predict true infection. These findings confirm the necessity of performing a QBC in dogs and cats with LUT signs to definitively diagnose a UTI before antimicrobial treatment.

Subclinical bacteriuria is defined by current guidelines as “the presence of bacteria in urine as determined by positive bacterial culture from a properly collected urine specimen, in the absence of clinical evidence of infectious urinary tract disease.”^10^ One study demonstrated that subclinical bacteriuria, present in 8.9% of the dogs in the study population, is nonprogressive and often transient despite lack of antimicrobial treatment.^12^ In this study, 24/239 (10.0%) dogs and cats were considered to have subclinical bacteriuria in accordance with the current definition, which includes both marked and minimal levels of bacterial growth on QBC.^10^ However, only 17 of these 24 had marked growth, representing 7.1% (17/239) of the study population. The ISCAID guidelines do not recommend treatment of subclinical bacteriuria except in rare circumstances.^10^

The present study had several limitations. Although the procedure for plating urine samples was standardized for laboratory personnel, there were numerous individuals that plated samples for this study. Individual variation could potentially impact results. Additionally, time from collection of urine sample to delayed plating at the reference laboratory was not the same for all samples. However, this reflects common circumstances in clinical practice. Although urine culture samples were shipped refrigerated in this study, this might not be feasible or standard procedure for all practices. It is important to note that these results could not be applied to samples that were shipped without temperature control. Another limitation is that the clinical samples were submitted by a variety of individuals. Hospital policy dictates that clean midstream catch urine samples be collected both midstream and in a clean or sterile container, followed by standardized transport to a sterile urine culture tube. However, given that a variety of students, clinicians, and technicians assist in collecting routine samples, we cannot verify adherence to protocol by all individuals. In a small number of cases, the presence of clinical signs, medication history, or both were unknown because of inadequate medical records. Lastly, there was a gap in sample collection over approximately 7 months, and sample collection was then resumed to increase sample size. Ideally samples would have been collected consecutively to eliminate temporal bias.

The results of this study indicate that with appropriate storage and transportation techniques, the immediate plating of urine samples for culture and sensitivity testing is unnecessary in the majority of cases. Voided samples are more likely to have discrepant results if sample processing is delayed, but such discrepancies are unlikely to have a clinical impact. Conversely, whereas cystocentesis samples with discordant results were more likely to alter clinical decisions, these discrepancies were uncommon.

## CONFLICT OF INTEREST DECLARATION

Authors declare no conflict of interest.

## OFF‐LABEL ANTIMICROBIAL DECLARATION

Authors declare no off‐label use of antimicrobials.

## INSTITUTIONAL ANIMAL CARE AND USE COMMITTEE (IACUC) OR OTHER APPROVAL DECLARATION

Authors declare no IACUC or other approval was needed.

## HUMAN ETHICS APPROVAL DECLARATION

Authors declare human ethics approval was not needed for this study.
